# Basal inferoseptal longitudinal strain deformation may indicate early cardiac involvement in wild-type carpal ATTR

**DOI:** 10.1093/eschf/xvag055

**Published:** 2026-02-16

**Authors:** Toshihiro Tsuruda, Tomomi Ota, Tamasa Terada, Hiroshi Nakada, Miyuki Ogata, Miyo Tanaka, Yosuke Suiko, Yunosuke Matsuura, Soichi Komaki, Kohei Moribayashi, Rina Yamada, Atsushi Yamashita, Keisuke Yamamoto, Kensaku Nishihira, Yoshisato Shibata, Koichi Kaikita

**Affiliations:** Cardiorenal Research Laboratory, Department of Hemo-Vascular Advanced Medicine, University of Miyazaki, 5200 Kihara, Kiyotake, Miyazaki 889-1692, Japan; Division of Cardiovascular Medicine and Nephrology, Department of Internal Medicine, University of Miyazaki, 5200 Kihara, Kiyotake, Miyazaki 889-1692, Japan; Division of Orthopaedic Surgery, Department of Medicine of Sensory and Motor Organs, University of Miyazaki, Miyazaki, Japan; Department of Radiology, University of Miyazaki, Miyazaki, Japan; Department of Radiology, University of Miyazaki, Miyazaki, Japan; Heart Center, University of Miyazaki, Miyazaki, Japan; Heart Center, University of Miyazaki, Miyazaki, Japan; Division of Cardiovascular Medicine and Nephrology, Department of Internal Medicine, University of Miyazaki, 5200 Kihara, Kiyotake, Miyazaki 889-1692, Japan; Division of Cardiovascular Medicine and Nephrology, Department of Internal Medicine, University of Miyazaki, 5200 Kihara, Kiyotake, Miyazaki 889-1692, Japan; Division of Cardiovascular Medicine and Nephrology, Department of Internal Medicine, University of Miyazaki, 5200 Kihara, Kiyotake, Miyazaki 889-1692, Japan; Division of Cardiovascular Medicine and Nephrology, Department of Internal Medicine, University of Miyazaki, 5200 Kihara, Kiyotake, Miyazaki 889-1692, Japan; Department of Radiology, University of Miyazaki, Miyazaki, Japan; Department of Pathology; Faculty of Medicine, University of Miyazaki, Miyazaki, Japan; Department of Cardiology, Miyazaki Medical Association Hospital, Miyazaki, Japan; Department of Cardiology, Miyazaki Medical Association Hospital, Miyazaki, Japan; Department of Cardiology, Miyazaki Medical Association Hospital, Miyazaki, Japan; Division of Cardiovascular Medicine and Nephrology, Department of Internal Medicine, University of Miyazaki, 5200 Kihara, Kiyotake, Miyazaki 889-1692, Japan

**Keywords:** Wild-type transthyretin amyloidosis, Carpal tunnel syndrome, Segmental longitudinal strain, Troponin-T, Strain echocardiography

## Abstract

**Background and Aims:**

Wild-type transthyretin cardiac amyloidosis (ATTRwt-CA) is now increasingly identified as a cause of heart failure in older adults. This study aimed to clarify the morphological and functional alterations of the left ventricle (LV) that define the early stage of this condition.

**Methods:**

We prospectively evaluated 81 patients diagnosed with wild-type ATTR (ATTRwt) amyloidosis (mean age 77 ± 6 years; 88% male), categorized into three groups based on myocardial uptake on radioactive pyrophosphate scintigraphy and histological confirmation: (i) carpal ATTR without cardiac involvement (Group 1, *n* = 13), (ii) asymptomatic cardiac involvement (Group 2, *n* = 10) and (iii) overt heart failure (Group 3, *n* = 58).

**Results:**

Compared with Group 3, Group 1 showed higher absolute global longitudinal strain (GLS) (median 19.0 [13.2–23.8]%, *P* < .001), a lower apical-sparing ratio (median 0.66 [0.55–1.04], *P* < .001) and lower brain natriuretic peptide (BNP) (median 13.5 [6–49] pg/mL, *P* < .001) and troponin-T concentrations (0.012 [0.006–0.022] ng/mL, *P* < .001), while the estimated glomerular filtration rate remained preserved (64 ± 9 mL/mL/1.73 m², *P* = .022). Segmental longitudinal strain (LS) differentiated Group 1 from Group 2, with basal inferoseptal LS significantly lower in patients with elevated troponin-T (> 0.014 ng/mL) than in those with lower values (13.9 ± 5.6% vs. 7.4 ± 1.8%, *P* = .046) in Group 1. A basal inferoseptal LS cutoff of 9.1% identified high troponin-T with an area under the curve (AUC) of 0.833 (*P* = .005), outperforming GLS (AUC 0.306, *P* = .217), BNP (AUC 0.667, *P* = .292), and LV ejection fraction (AUC 0.556, *P* = .743).

**Conclusions:**

Basal inferoseptal LS impairment may indicate early cardiac involvement in individuals with carpal tunnel syndrome carrying ATTRwt deposits.

## Introduction

Transthyretin-derived cardiac amyloidosis (ATTR-CA) is a progressive and frequently underdiagnosed type of heart failure caused by the extracellular accumulation of misfolded transthyretin (TTR) protein within the myocardium.^1^ Wild-type ATTR-CA (ATTRwt-CA) represents about 15% of heart failure with preserved ejection fraction (HFpEF)^2–4^ and 11% of heart failure with reduced ejection fraction (HFrEF)^5^ among older adults.

The main clinical difficulty lies in the early identification of amyloid deposits to manage patients who have or are at risk of developing ATTRwt-CA, since prompt treatment provides substantial clinical benefit.^6–8^ Although early-stage ATTRwt-CA has been described by lower N-terminal pro-B-type natriuretic peptide (NT-proBNP) levels, preserved estimated glomerular filtration rate (eGFR) and minimal diuretic requirements at diagnosis,^7,9^ these staging criteria have limitations, as clinical practice often detects incidental ATTR deposits in extra-cardiac sites such as the carpal tunnel,^10^ along with silent cardiac involvement that does not yet necessitate diuretics. Based on our earlier findings,^11^ we hypothesize that deformation in the basal inferoseptal segment may act as an early sign of morphological change indicating disease advancement. This study aimed to evaluate echocardiographic segmental features together with electrocardiographic and biochemical parameters in patients with ATTRwt amyloidosis, classified into three clinical groups: (1) carpal ATTR without cardiac involvement, (2) asymptomatic cardiac involvement and (3) overt heart failure.

## Methods

### Study population

**Figure 1:**
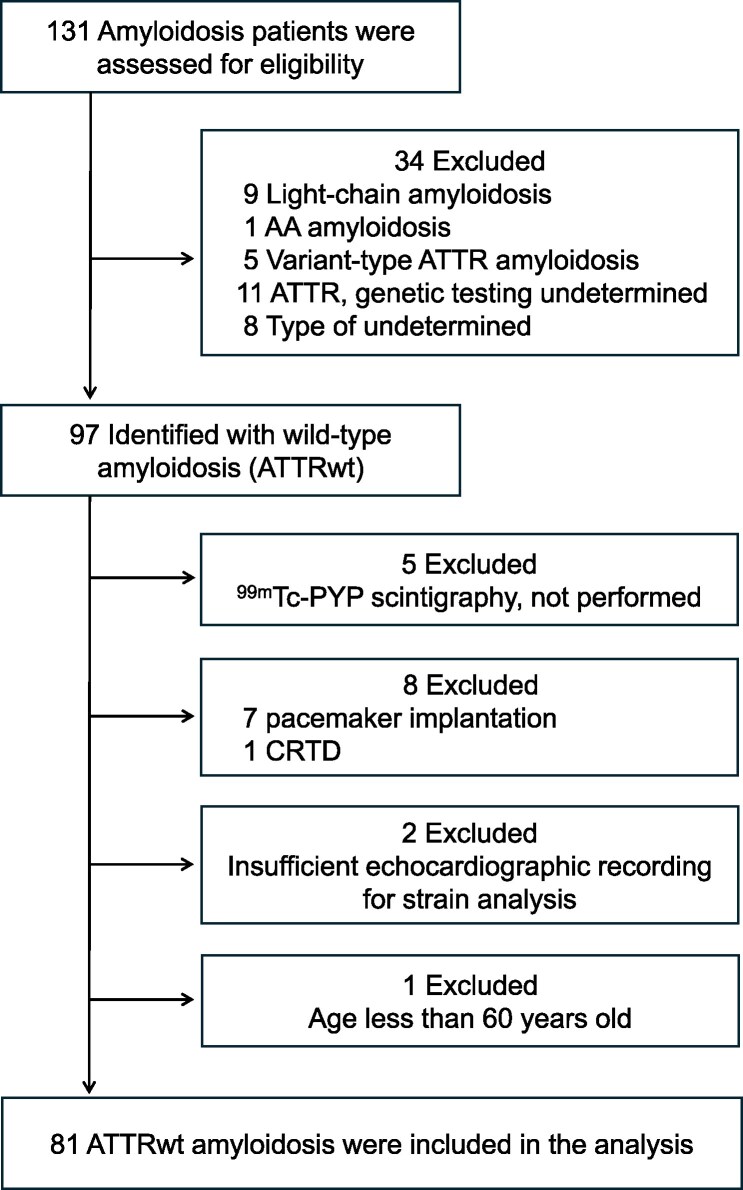
Study enrolment overview. ATTR, amyloid transthyretin; wt, wild-type; ^99m^Tc-PYP, ^99m^Techneisum pyrophosphate; CRTD, cardiac resynchronization therapy-defibrillator

This prospective single-centre study included 81 consecutive patients diagnosed with ATTRwt amyloidosis, with or without cardiac involvement, between September 2018 and December 2024, who were admitted to Miyazaki University Hospital, Japan. An overview of the study enrolment is presented in *Figure 1*. Ethical approval was granted by the University of Miyazaki Institutional Committee (Protocol number, O-0651). All patients received care in line with the Declaration of Helsinki and gave informed consent for the anonymous use of their data in publication.

### Criteria definition

**Figure 2:**
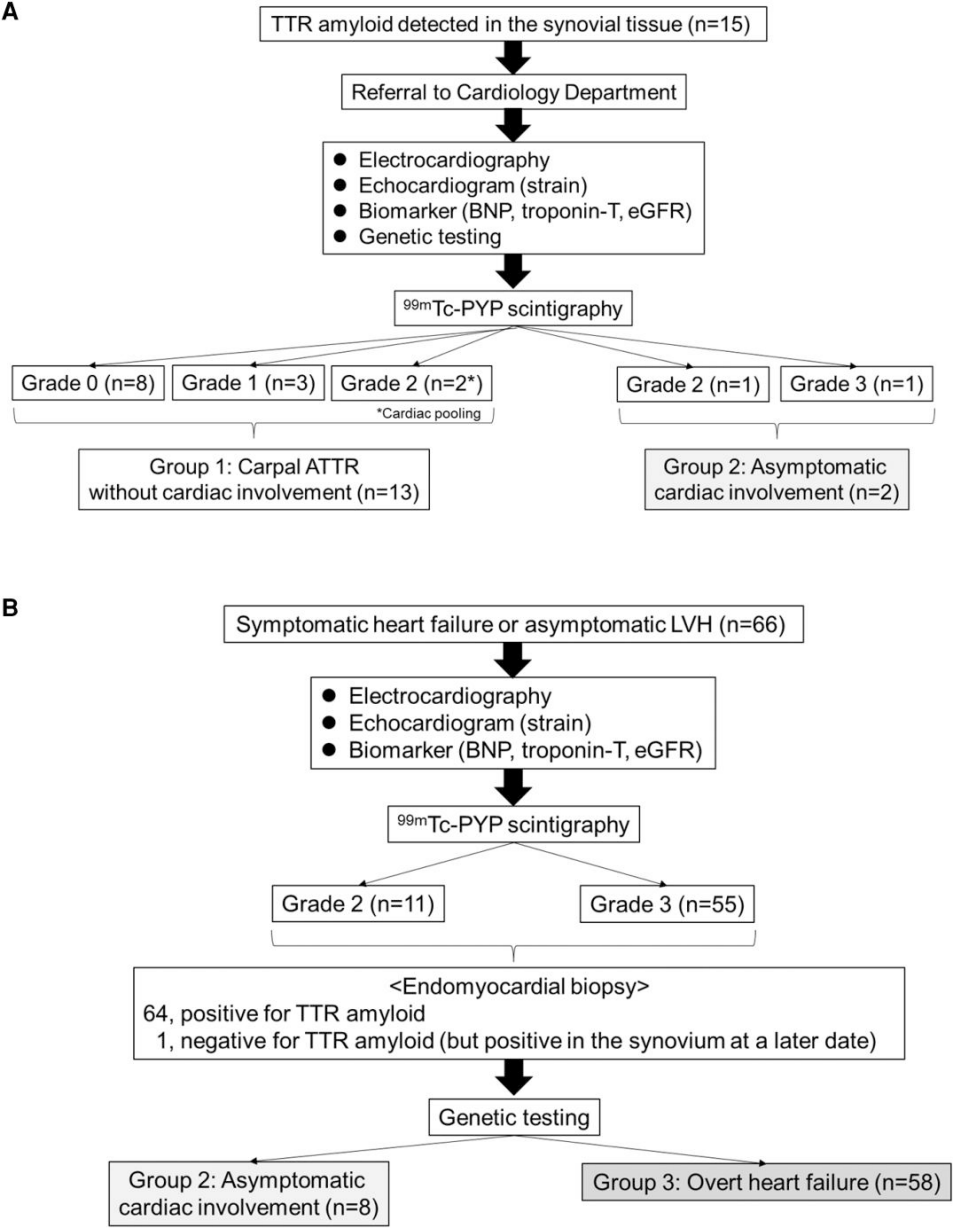
Classification diagram based on ^99m^Techneisum pyrophosphate (^99m^Tc-PYP) scintigraphy and cardiac symptoms. (*A*) Detection of transthyretin-derived amyloid in synovial tissue from patients with carpal tunnel syndrome. (*B*) Detection of transthyretin-derived amyloid in myocardial tissues in patients with symptomatic cardiomyopathy or asymptomatic left ventricular hypertrophy (LVH). Abbreviations: TTR, transthyretin; BNP, brain natriuretic peptide; eGFR, estimated glomerular filtration rate; ATTR, amyloid transthyretin

This study employed two pathways for patient recruitment: (i) *synovial biopsy* was conducted in patients with carpal tunnel syndrome who had no cardiac symptoms at the time of surgery; if TTR amyloid was identified in the tissue, orthopaedic surgeons referred the patient to the cardiology department (*Figure 2A*); (ii) *cardiac biopsy* was performed in patients who had symptomatic heart failure or asymptomatic left ventricular (LV) hypertrophy with Perugini Grade 2 or 3 myocardial uptake on a ^99m^Technesium-pyrophosphate (^99m^Tc-PYP) radionuclide scan *(Figure 2B*). Based on ^99m^Tc-PYP scintigraphy findings and cardiac symptoms, patients were categorized into three groups. Group 1, carpal ATTR without cardiac involvement, included patients without cardiac symptoms but with confirmed TTR amyloid deposition in synovial tissue and one of the following scan results: Perugini Grade 0 or 1 myocardial uptake on ^99m^Tc-PYP scintigraphy, or Grade 2 uptake with residual blood pool activity in the heart.^12^ Group 2, asymptomatic cardiac involvement, was defined by the absence of cardiac symptoms with Perugini Grade ≥2 myocardial uptake on ^99m^Tc-PYP scintigraphy and confirmed TTR amyloid deposition in the myocardium or synovium. Group 3, overt heart failure, was defined as symptomatic heart failure with histological evidence of TTR amyloid and Perugini Grade ≥2 myocardial uptake on ^99m^Tc-PYP scintigraphy. Inclusion criteria required patients to be aged ≥60 years and to have no pathogenic TTR gene mutation. Exclusion criteria were the presence of a monoclonal protein detected by serum-free light chain assay or serum and urine immunofixation,^7^ other forms of amyloidosis (e.g. AL amyloidosis, variant-type ATTR amyloidosis, AA amyloidosis), lack of ^99m^Tc-PYP scintigraphy, amyloid typing or genetic testing, a history of myocardial infarction or poor-quality echocardiographic images unsuitable for strain analysis. Comorbid conditions such as hypertension, diabetes mellitus and dyslipidaemia were defined as previously described.^11^ Medications were recorded at the time of echocardiography. Disease severity was staged according to the National Amyloidosis Centre (NAC), London: Stage I was defined as NT-proBNP ≤3000 pg/mL and eGFR ≥45 ml/min; Stage III was NT-proBNP >3000 pg/mL and eGFR <45 ml/min; and Stage II included patients not meeting Stage I or III criteria.^9^ NAC ATTR Stage I was further subdivided: Stage Ia was defined as a furosemide equivalent diuretic dose <0.75 mg/kg and NT-proBNP ≤500 pg/mL or ≤1000 pg/mL in patients with atrial fibrillation, and Stage Ib included all other Stage I patients.^7^ The conversion used for other loop diuretics was furosemide 20 mg = azosemide 30 mg.^13^

### Histology and immunohistochemical staining

Endomyocardial biopsy samples taken from the right interventricular septum were fixed in 10% formalin and embedded in paraffin. The presence of ATTR fibrils was identified by staining with Congo red dye or Direct Fast Scarlet.^11^ Stained sections were examined under polarized light, and immunohistochemical staining with a TTR_115–124_ rabbit polyclonal antibody was performed to confirm the type of amyloid fibrils.^14^ Slides were scanned at ×40 magnification using an Olympus BX53F microscope (Olympus, Tokyo, Japan). The area with positive immunohistochemical staining for TTR was measured as a percentage of the total area of the endomyocardial samples using WinROOF 2018 (Mitani Co., Tokyo, Japan).

### Genetic testing

The presence or absence of a mutation was confirmed by amplifying and sequencing exons 1–4 of the TTR gene, using DNA extracted from whole blood with a polymerase chain reaction assay.^11^

### 99mTc-PYP radionuclide scintigraphy

Myocardial uptake of ^99m^Tc-PYP was assessed 3 h after administration of 555–740 MBq of ^99m^Tc-PYP, using the Perugini visual scoring system (Grades 0–3).^15^ The exact location of radiotracer uptake within the myocardium was verified using SPECT/CT imaging.^16^

### Biochemical analysis

Blood samples were collected immediately before echocardiography to measure eGFR, brain natriuretic peptide (BNP) levels (Abbott) and high-sensitivity troponin-T (Roche Diagnostics). BNP values were converted to NT-proBNP using Convers.App for BNP and NT, created by Dr. Yoshihiko Saito.^17^

### 12-lead Electrocardiogram

Electrocardiographic measurements followed standard definitions^18,19^: low QRS voltage was defined as ≤5 mm (0.5 mV) in all peripheral leads or ≤1 mV in all precordial leads; a pseudo-infarction pattern was defined as pathological Q waves (1/4 R amplitude) or QS waves on two consecutive leads without previous ischaemic heart disease; and atrioventricular conduction delays included any degree of atrioventricular block, while intraventricular conduction delays were classified as complete (QRS duration ≥0.12 s) or incomplete (QRS duration 0.1–0.12 s) right or left bundle branch block.

### Echocardiographic assessment

We used the EPIQ CVx system (Philips, Amsterdam, Netherlands) and the Vivid E95 ultrasound system (GE Healthcare, Horten, Norway) to obtain standard echocardiographic data as previously described.^11^ For two-dimensional speckle-tracking echocardiography, apical four-, three- and two-chamber views were acquired and stored in a cine-loop format. These images were triggered to the QRS complex over two cardiac cycles, with frame rates set between 40 and 80 frames per second. Longitudinal strain (LS) was measured using AutoStrain LV/RV/LA-automated strain analysis on the Philips EPIQ CVx system. For images recorded with the Vivid E95 system, offline analysis was carried out using vendor-independent software (TOMTEC ARENA Imaging Systems GmbH) to avoid inter-vendor variability in global and segmental LS values.^20^ Endocardial borders of the LV were traced in each long-axis view, assessing both end-diastolic and end-systolic frames. Manual tracking started approximately 1 cm below the aortic annulus and excluded the mitral valve annulus. In the apical three-chamber view, the sigmoid septum segment was excluded. LS values were reported as absolute measures for global longitudinal strain (GLS),^11,21^ and for regional LS at the basal, midventricular and apical levels. An additional segmental analysis applied an 18-segment model based on the 2D speckle-tracking data, with the results illustrated in a bull’s-eye map.^11^ For patients with atrial fibrillation, a single beat with nearly equal preceding R-R intervals was chosen for analysis. Five designated sonographers performed all image acquisitions and analyses blinded to the biochemical data. To assess apical sparing, the relative regional strain index (RapLSI) was calculated as the average LV LS of the apical segments divided by the average LV LS of the mid and basal segments.^22^

### Statistical analysis

The normality of the data was tested using the Shapiro–Wilk test. Continuous variables were reported as mean ± standard deviation for parametric data or as median (25th and 75th percentiles) for non-parametric data, while categorical variables were shown as absolute counts (percentage). Differences in categorical variables among the three groups were analysed using Pearson’s chi-squared test or Fisher’s exact test. For comparing two continuous variables, either the Student’s *t*-test or the Mann–Whitney *U* test was applied. For comparisons of a continuous variable across three independent groups, one-way ANOVA (parametric) followed by Tukey’s post-hoc test or the Kruskal–Wallis test (non-parametric) followed by Bonferroni correction was used to examine differences. Pearson’s correlation coefficient (r) was used to assess the linear association between LS measured by echocardiography and amyloid deposition in the endomyocardial samples. The intraclass correlation coefficient (ICC) was calculated to assess the reproducibility of the 2D speckle-tracking echocardiographic measurements performed by five sonographers.^23^ Receiver operating characteristic (ROC) curve analysis was used to examine the discriminative performance of GLS, LVEF, BNP and basal inferoseptal LS in predicting elevated troponin-T levels. The area under the curve (AUC) and corresponding confidence intervals were calculated, and Youden’s index was used to identify the optimal cutoff points. A probability value of <.05 was considered statistically significant. All graphs and statistical analyses were conducted using GraphPad Prism 8.0 (GraphPad Software, La Jolla, CA, USA) and SPSS Statistics version 29 (IBM Corporation, Armonk, New York).

## Results

### Baseline characteristics

The characteristics of the 81 patients with ATTRwt amyloidosis included in this study are summarized in *Table 1*, along with comparisons across the three groups. Patients with carpal ATTR without cardiac involvement (Group 1) were younger, had a higher body mass index, were more likely to have hypertension and underwent coronary interventions less frequently than those with asymptomatic cardiac involvement (Group 2) or overt heart failure (Group 3). On electrocardiography, Group 1 showed no low voltage or atrial flutter/fibrillation and had a lower prevalence of first-degree atrioventricular block compared with Group 2 or Group 3. Myocardial radioactive pyrophosphate uptake of Grade ≥2 on scintigraphy, which is highly specific for diagnosing ATTRwt-CA,^24^ was observed in all patients in Group 2 and Group 3. In contrast, two patients (15%) in Group 1 demonstrated Grade 2 uptake, which was attributed to cardiac blood pooling. Neither Group 1 nor Group 2 patients received loop diuretics, mineralocorticoid receptor antagonists or sodium–glucose cotransporter 2 inhibitors, which were more commonly prescribed in Group 3. In this cohort, 100% of Group 1 and 60% of Group 2 were classified as NAC Stage Ia, compared with 24% of Group 3. Group 1 also showed less hypertrophy, preserved systolic function and lower E/e’ compared with Group 2 or Group 3 (*Table 2*).

**Table 1:** Demographics characteristics of the wild-type ATTR amyloidosis cohort

	All patients (*n* = 81)	Group 1 (*n* = 13)	Group 2 (*n* = 10)	Group 3 (*n* = 58)	*P*-value
Age at diagnosis (years)	77 ± 6	73 ± 4^#^	74 ± 6	78 ± 6	.006
Male sex, *n* (%)	71 (88)	9 (69)	9 (90)	53 (91)	.088
Body mass index, kg/m^2^	23 [22, 26]	26 [24, 28]^##^	23 [22, 25]	23 [20, 26]	.007
Comorbidities					
Hypertension, *n* (%)	55 (68)	12 (92)	9 (90)	34 (59)	.018
Diabetes mellitus, *n* (%)	21 (26)	5 (38)	2 (20)	14 (24)	.511
Dyslipidaemia, *n* (%)	28 (35)	7 (54)	4 (40)	17 (29)	.226
Carpal tunnel syndrome, *n* (%)	43 (54)	13 (100)	7 (70)	23 (40)	<.001
Severe aortic valve stenosis, *n* (%)	1 (1)	0 (0)	0 (0)	1 (2)	.818
Coronary interventions, *n* (%)	14 (17)	1 (8)	1 (10)	12 (21)	<.001
SBP (mmHg)	125 ± 18	140 ± 20^##^	132 ± 13	120 ± 17	<.001
DBP (mmHg)	74 ± 12	80 ± 9	80 ± 10	71 ± 13	.017
Pulse rate (bpm)	73 ± 13	69 ± 8	74 ± 14	74 ± 14	.540
Electrocardiogram					
Low voltage, *n* (%)	17 (21)	0 (0)	0 (0)	17 (29)	.014
AF/flutter, *n* (%)	23 (28)	0 (0)	1 (10)	22 (38)	.038
First atrioventricular conduction delay, *n* (%)	32 (40)	5 (38)	4 (40)	23 (40)	.019
LBBB, *n* (%)	13 (16)	0 (0)	0 (0)	13 (22)	.152
RBBB, *n* (%)	12 (15)	1 (8)	3 (30)	8 (14)	.595
IBBB, *n* (%)	22 (27)	2 (15)	1 (10)	19 (33)	.421
Pseudo-infarct pattern, *n* (%)	20 (25)	2 (14)	3 (30)	15 (26)	.670
Biopsy sites					<.001
Heart, *n* (%)	65 (80)	0 (0)	8 (80)	57 (98)	
Synovium, *n* (%)	16 (20)	13 (100)	2 (20)	1 (2)	
^99m^Tc-PYP (grade, 0/1/2/3), *n*	8/3/14/56	8/3/2/0	0/0/4/6	0/0/8/50	<.001
Medications					
Loop diuretic, *n* (%)	41 (51)	0 (0)	0 (0)	41 (71)	<.001
ACEi/ARB, *n* (%)	38 (47)	6 (46)	6 (60)	26 (45)	.673
MRA, *n* (%)	32 (40)	0 (0)	0 (0)	32 (55)	<.001
Beta blocker, *n* (%)	27 (33)	0 (0)	1 (10)	26 (45)	.002
ARNI, *n* (%)	8 (10)	0 (0)	0 (0)	8 (14)	.172
SGLT2i, *n* (%)	29 (36)	1 (8)	1 (10)	27 (47)	.006
sGC stimulator, *n* (%)	2 (2)	0 (0)	0 (0)	2 (3)	.666
NAC staging (Ia/Ib/II/III)	33/28/17/3	13/0/0/0	6/3/1/0	14/25/16/3	<.001

SBP, systolic blood pressure; DBP, diastolic blood pressure; AF, atrial fibrillation; LBBB, left bundle branch block; RBBB, right bundle branch block; IBBB, incomplete bundle branch block; ^99m^Tc-PYP, ^99m^Tc-pyrophosphate; ATTR, amyloid transthyretin; BNP, brain natriuretic peptide; eGFR, estimated glomerular filtration rate; NAC, National Amyloidosis Centre; ACEi, angiotensin-converting enzyme inhibitor; ARB, angiotensin II type 1 receptor blocker; MRA, mineralocorticoid receptor antagonist; ARNI, angiotensin receptor neprilysin inhibitor; SGLT2i, sodium–glucose cotransporter 2 inhibitor; sGC, soluble guanylate cyclase.

Group 1 indicates carpal ATTR without cardiac involvement; Group 2 represents asymptomatic cardiac involvement; Group 3 indicates overt heart failure. Continuous data are shown as mean ± SD or median [25th, 75th percentiles], and categorical variables are presented as counts (percentages). Differences among the three groups were analysed using one-way ANOVA (parametric) with Tukey’s adjustment for post-hoc pairwise comparisons, or the Kruskal–Wallis (non-parametric) with Bonferroni correction. ^#^*P* < .05, ^##^*P* < .01 (Group 1 vs. Group 3). Categorical variables across groups were tested with the Pearson’s chi-squared test.

**Table 2:** Echocardiographic parameters in the wild-type ATTR amyloidosis cohort

	All patients	Group 1	Group 2	Group 3	*P*-value
Echocardiogram	(*n* = 81)	(*n* = 13)	(*n* = 10)	(*n* = 58)	
IVSTd (mm)	16 [13, 18]	11 [11, 12] ^*, ###^	15 [12, 16]	17 [16, 18]	<.001
LVPWTd (mm)	15 [12, 17]	11 [10, 12] ^**, ###^	15 [12, 17]	16 [14, 17]	<.001
LVEF (%)	52 ± 10	63 ± 5 ^###^	56 ± 6^$^	48 ± 10	<.001
E/e’	16 ± 5 (*n* = 78)	10 ± 4 ^###^	14 ± 3 (*n* = 8)	17 ± 5 (*n* = 57)	<.001
Regional LS (%)					
Base	7 [4, 11]	17 [16, 23] ^*, ###^	9 [7, 12] ^$$^	5 [3, 8]	<.001
Mid-ventricle	9 [6, 13]	16 [15, 21] ^**, ###^	9 [13, 18]	8 [5, 11]	<.001
Apex	20 ± 5	24 ± 3 ^*, ##^	19 ± 5	19 ± 5	.008

IVSTd, interventricular septal thickness at end-diastole; LVPWTd, left ventricular posterior wall thickness at end-diastole; LVEF, left ventricular ejection fraction; LS, longitudinal strain (expressed as absolute values).

Group 1 indicates carpal ATTR without cardiac involvement; Group 2 represents asymptomatic cardiac involvement; Group 3 indicates overt heart failure. Continuous variables are presented as mean ± SD or median [25th, 75th percentiles], and categorical variables as counts (percentages). One-way ANOVA (parametric) with Tukey’s adjustment for post-hoc comparisons or the Kruskal–Wallis test (non-parametric) with Bonferroni correction were applied to evaluate differences among the three groups. *P < .05, **P < .01 (Group 1 vs. Group 2); ^##^*P* < .01, ^###^*P* < .001 (Group 1 vs. Group 3); ^$^*P* < .05, ^$$^*P* < .01 (Group 2 vs. Group 3). Categorical variables were compared with Pearson’s chi-squared test.

### GLS, apex-to-base ratio and biomarkers in blood

**Figure 3:**
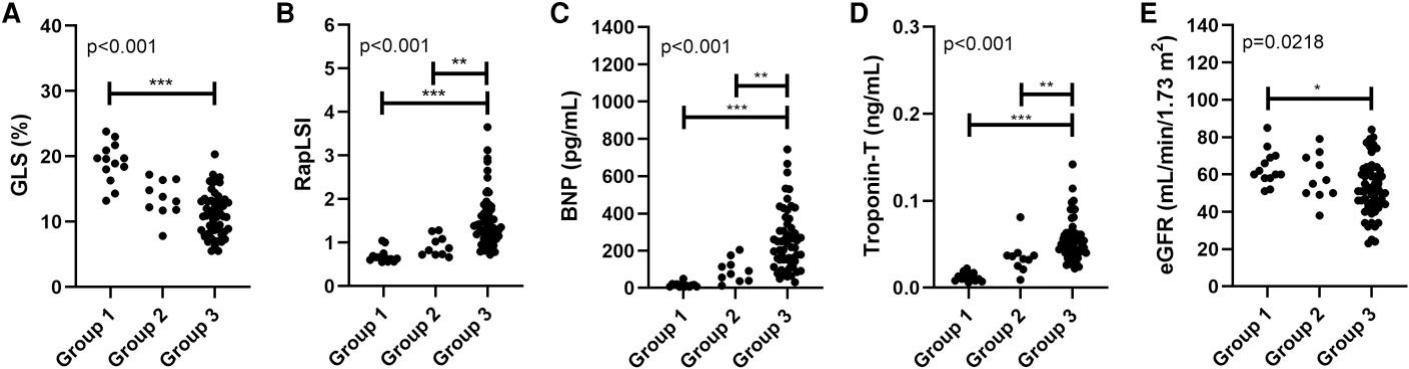
Imaging and blood biomarkers in wild-type ATTR amyloidosis. (*A*) GLS, global longitudinal strain; (*B*) RapLSI, relative apical longitudinal strain index; (*C*) BNP, brain natriuretic peptide; (*D*) Troponin-T; and (*E*) eGFR, estimated glomerular filtration rate, are shown as dot plots. *P < .05, **P < .01, ***P < .001, determined by the Kruskal–Wallis test with Bonferroni correction (*A–D*) or one-way ANOVA with Tukey’s adjustment (*E*). LS values shown as absolute numbers. Group 1, carpal ATTR without cardiac involvement (*n* = 13); Group 2, asymptomatic cardiac involvement (*n* = 10); Group 3, overt heart failure (*n* = 58)

The median GLS was 19% in Group 1 and decreased to 13% in Group 2 and 11% in Group 3 (*Figure 3A*). The apical sparing pattern, indicated by a RapLSI value >1, was present in 15% of Group 1 but appeared more frequently in Group 2 (40%) and Group 3 (79%) (*Figure 3B*). Biomarkers such as BNP and troponin-T were lower in Group 1 than in Groups 2 or 3 (*Figures 3C* and *D*). In contrast, Group 1 maintained a preserved glomerular filtration rate compared with Group 2 or 3 (*Figure 3E*). None of these markers distinguished Group 1 from Group 2.

### Segmental LS analysis

**Figure 4:**
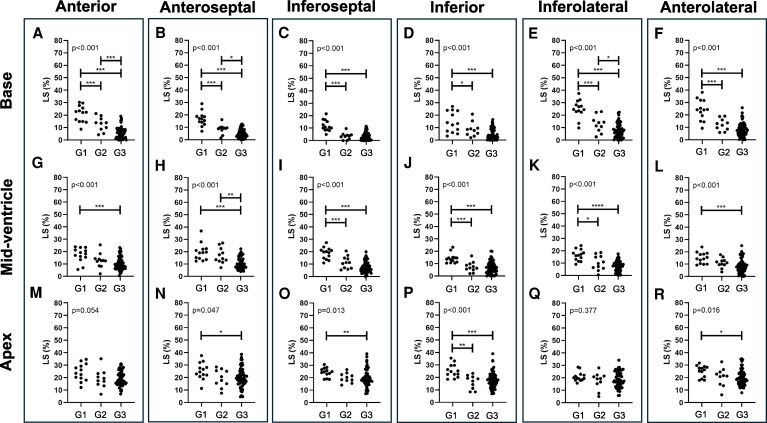
Comparison of longitudinal strain (LS) in six myocardial segments at the base (*A–F*), mid-ventricle (*G–L*) and apex (*M–R*). Groups: G1 (carpal ATTR without cardiac involvement, *n* = 13), G2 (asymptomatic cardiac involvement, *n* = 10), G3 (overt heart failure, *n* = 58). Single measurements by five sonographers are shown as dot plots. *P < .05, **P < .01, ***P < .001 using one-way ANOVA with Tukey’s post-hoc adjustment (*A–J*) or Kruskal–Wallis with Bonferroni correction (*K–R*). LS shown as absolute values. G1, Group 1; G2, Group 2; G3, Group 3

As shown in *Figure 4*, segmental LS worsened progressively with advancing cardiac involvement across all basal and mid-ventricular segments; significant differences between Group 1 and Group 2 were seen in all basal segments (A–F), as well as in the inferoseptal, inferior and inferolateral segments at the mid-ventricular level (I, J, K) and the inferior segment at the apical level (P).

### Diagnostic performance of segmental strain analysis in carpal ATTR without cardiac involvement

**Figure 5:**
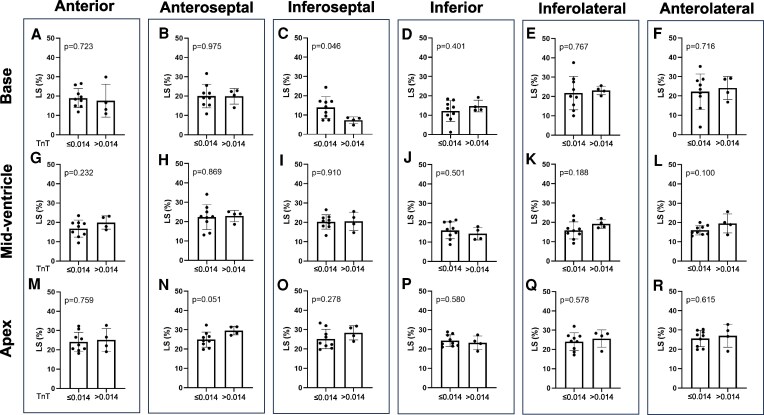
Segmental longitudinal (LS) strain and serum troponin-T in carpal ATTR without cardiac involvement. Segmental LS for six myocardial segments at the base (*A–F*), mid-ventricle (*G–L*) and apex (*M–R*) in Group 1 (*n* = 13). Average segmental LS values measured by five sonographers shown as dot plots (mean ± SD), analysed using Student’s *t*-test. LS expressed as absolute values. Abbreviation: TnT, troponin-T (ng/mL)

**Figure 6:**
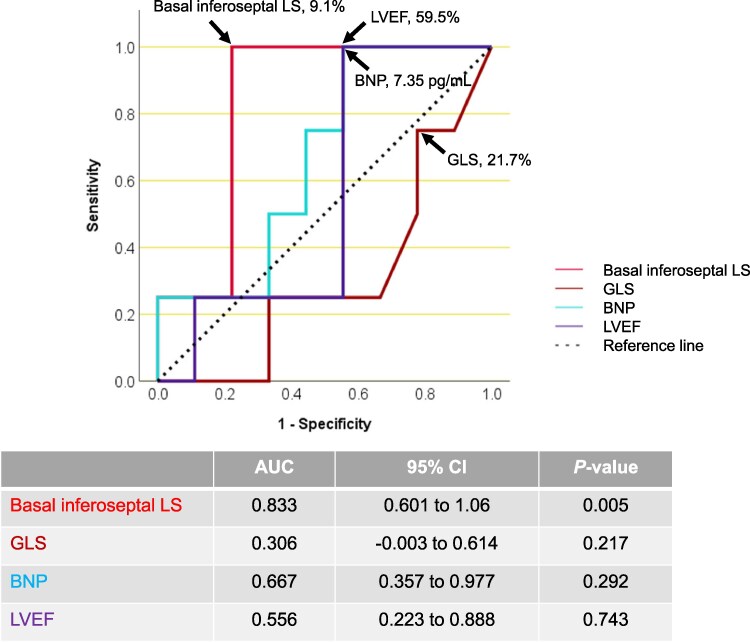
Receiver operating characteristic (ROC) analysis. ROC curves of basal inferoseptal longitudinal strain (LS), global LS (GLS), brain natriuretic peptide (BNP) and left ventricular ejection fraction (LVEF) to predict troponin-T > .014 ng/mL. Abbreviations: AUC, area under the curve; CI, confidence interval

Serum troponin-T is known to detect myocardial injury in ATTRwt-CA,^25^ so we investigated whether inferoseptal LS might be related to serum troponin-T levels. In Group 1, 4 of the 13 patients had elevated troponin-T levels exceeding 0.014 ng/mL. *Figure 5* shows that only inferoseptal LS, averaged across measurements by five sonographers, was significantly different when using the troponin-T threshold of 0.014 ng/mL. *Figure 6* displays the ROC analyses evaluating the predictive performance of GLS, LVEF, BNP and basal inferoseptal LS for identifying troponin-T levels greater than 0.014 ng/mL. A basal inferoseptal LS cutoff of 9.1% achieved a sensitivity of 100% and specificity of 78%, whereas a GLS cutoff of 21.7% gave a sensitivity of 75% and a specificity of 22%. A BNP cutoff of 7.35 pg/mL showed a sensitivity of 100% and specificity of 44%, and an LVEF cutoff of 59.5% provided a sensitivity of 100% and specificity of 22%.

## Discussion

As opportunities to identify patients with ATTRwt-CA before the onset of heart failure symptoms have increased, more individuals are now diagnosed at an early stage. This study aimed to describe the morphological and functional characteristics of the LV in patients with ATTR deposits limited to the carpal synovium, to better understand risk stratification.

Amyloid fibril deposition can be visualized by conventional echocardiography as marked LV hypertrophy with a restrictive filling pattern, but this may go undetected in earlier phases.^24,26^ Reflecting this, patients with carpal ATTR without cardiac involvement showed less hypertrophy than those with asymptomatic cardiac involvement or overt heart failure. Moreover, electrocardiographic parameters, BNP, troponin-T and eGFR levels did not distinguish patients with ATTR deposits restricted to the carpal synovium from those with silent cardiac involvement. LS measured at the endocardial border can identify subtle LV deformation,^22^ and our findings align with the previous work showing that GLS and the apical sparing ratio are useful in describing advanced ATTRwt-CA^22,27^; however, these measures appear less informative for identifying early stages. The results of this study indicate that segmental LS analysis, particularly in the basal slice, may aid in distinguishing Group 1 from Group 2. We observed that elevated troponin-T can occur in Group 1 patients and was linked to LS reduction in the basal inferoseptum. To evaluate this clinically, we conducted ROC analyses and found that reduced LS (cutoff, 9.1%) in the basal inferoseptum predicted higher troponin-T levels more effectively than GLS, BNP or LVEF. Our earlier report showed that pronounced LS impairment in the basal inferoseptum was related to increased extracellular volume fraction as measured by cardiac MRI.^11^ The findings in this study may similarly reflect the extent of amyloid infiltration and resulting myocardial injury in a localized area, even in the absence of hypertrophy and overt heart failure.^11,28^ Consistently, LS reduction in the basal inferoseptal segment was not associated with the amount of amyloid deposition in right ventricular septum samples among Group 2 and Group 3 patients (*n* = 59, r = −0.095, *P* = .476). Ichimata et al.^29^ reported that, in early cases based on forensic autopsy findings, the basal ventricular septum is the region with the most pronounced amyloid deposition. Preferential amyloid build-up in the basal inferoseptum likely results from a combination of nonuniform mechanical stress, reduced amyloid clearance and the local composition of the extracellular matrix.^30–33^ Although this study’s sample size is limited and the results should be interpreted with caution, the findings suggest that reduced basal inferoseptal LS strain may be linked to early cardiac involvement in patients with carpal tunnel syndrome who carry ATTR deposits.

This study highlights two clinically relevant points. First, individuals at higher risk—such as asymptomatic elderly patients with carpal tunnel syndrome—may benefit from regular 2D speckle-tracking echocardiographic monitoring. The pattern of progressive deposition beginning in this susceptible region could be used to develop early imaging markers and guide decisions on surveillance or treatment in at-risk patients. In particular, this study indicates that segmental LS analysis in patients with confirmed ATTR deposits in carpal tunnel syndrome, or following carpal tunnel release, may help detect early cardiac progression in a timely manner. Second, our findings suggest that the role of ^99m^Tc-PYP scintigraphy for patients in the earliest stages still needs to be validated. In this study, we used ^99m^Tc-PYP scintigraphy to classify ATTR deposition as either isolated to the carpal tunnel or not.^34^ This study raises the question of how much myocardial amyloid infiltration is needed to produce detectable radionuclide uptake and indicates that false-negative ^99m^Tc-PYP scintigraphy scans can occur in early-stage ATTRwt-CA when amyloid deposits are still minimal.^35^ Negative scans with significant residual blood pool activity are often seen with Perugini Grade 1 or 2 findings.^36^ Therefore, relying solely on the PYP grade may oversimplify the complex progression through the three clinical stages.

Carpal tunnel syndrome caused by ATTR may precede cardiac involvement,^37^ and this was supported in our study by the heart being less structurally and functionally advanced in these patients. However, 30% of patients with asymptomatic cardiac involvement and 60% of those with overt heart failure did not present with carpal tunnel syndrome or a prior history of carpal tunnel release. As a result, it remains unclear how much ATTR deposition in the synovial tissue will eventually lead to clinical heart failure.^38^ The delay between the initial onset of heart failure and an accurate diagnosis can be unacceptably long and significantly affects survival.^39^ Currently, disease-modifying treatments (e.g. tafamidis, acoramidis and vutrisiran) are only available to symptomatic patients on diuretics.^40^ The survival benefit is the greatest in patients who are NYHA class I or II compared with those in class III.^41^ Further research is needed to clarify whether a watchful waiting period, during which patients are not yet eligible for disease-modifying therapy, is truly harmless.

### Limitations

This was a single-centre study with a limited sample size, especially for patients with ATTR deposition restricted to the carpal synovium and those with asymptomatic cardiac involvement. The two recruitment pathways used may have introduced selection bias. Coronary angiography and cardiac biopsy were not performed for Group 1 patients; therefore, we could not fully exclude the possibility that undiagnosed coronary artery disease or other cardiomyopathies influenced LS or troponin-T levels. Moreover, patients in Group 1 were defined by Grade 1 or Grade 2 (with cardiac pooling) myocardial uptake on ^99m^Tc-PYP scintigraphy; however, the absence of cardiac ATTR deposits was not confirmed by endomyocardial biopsy. The aim of this study was to characterize LV structural and functional features in this preclinical or early-stage cohort. Performing invasive procedures such as biopsy was therefore not ethically acceptable in the absence of cardiac involvement. Endomyocardial biopsy samples only a small region of the right interventricular septum. Because amyloid deposition can be patchy or regionally variable, there is a risk of sampling error; while biopsies can confirm the presence of amyloid, they provide limited information about the total amyloid burden.^29,42^ It remains uncertain whether deformation of a single inferoseptal segment is sensitive enough to detect early-stage amyloid cardiomyopathy. The ICC for single measurements of LS—most notably at the apex slice and basal inferoseptum—was moderate (ICC = 0.779), while the ICC for average measures was high (ICC = 0.946) (Supplementary Table S1). The basal inferoseptum often appears hypokinetic even in healthy individuals, due to nonuniformity of circumferential and longitudinal myocardial strain.^32^ In hypertrophic cardiomyopathy, segmental LS impairment strongly correlates with the degree and distribution of myocardial hypertrophy, with notable LS reductions at the basal anteroseptum (10.9%) and inferoseptum (11.5%) when using an 18-segment LV model.^43^ For this reason, we used average GLS and segmental LS values in the analyses to assess their predictive value for troponin-T status, minimizing variability and maintaining consistency. The reliability of segmental LS measurements could be enhanced through standardized image acquisition protocols and consistent evaluation of the intra- and interoperator variability. Further multicentre studies with larger cohorts are needed to confirm these findings and support their broader applicability.

## Conclusions

This study indicates that basal inferoseptal LS deformation may serve as a sensitive marker for the early detection of cardiac involvement in patients with wild-type carpal ATTR. The pronounced LS impairment in the basal inferoseptum may reflect myocardial damage resulting from regional amyloid deposition.

## Supplementary Material

xvag055_Supplementary_Data

## Data Availability

The primary data supporting the findings of this study are included in the main article and the Supplementary materials. Further information is available from the corresponding author upon reasonable request.
