# Supplementary material for: Basal inferoseptal longitudinal strain deformation may indicate early cardiac involvement in wild-type carpal ATTR
Source: ESC Heart Fail. 2026 Feb 16;13(2):xvag055. doi: 10.1093/eschf/xvag055 (PMC13036830; doi:10.1093/eschf/xvag055)
Supplement: xvag055_Supplementary_Data [file xvag055_supplementary_data.zip › Suppl Table S1.pptx]

## Slide 1
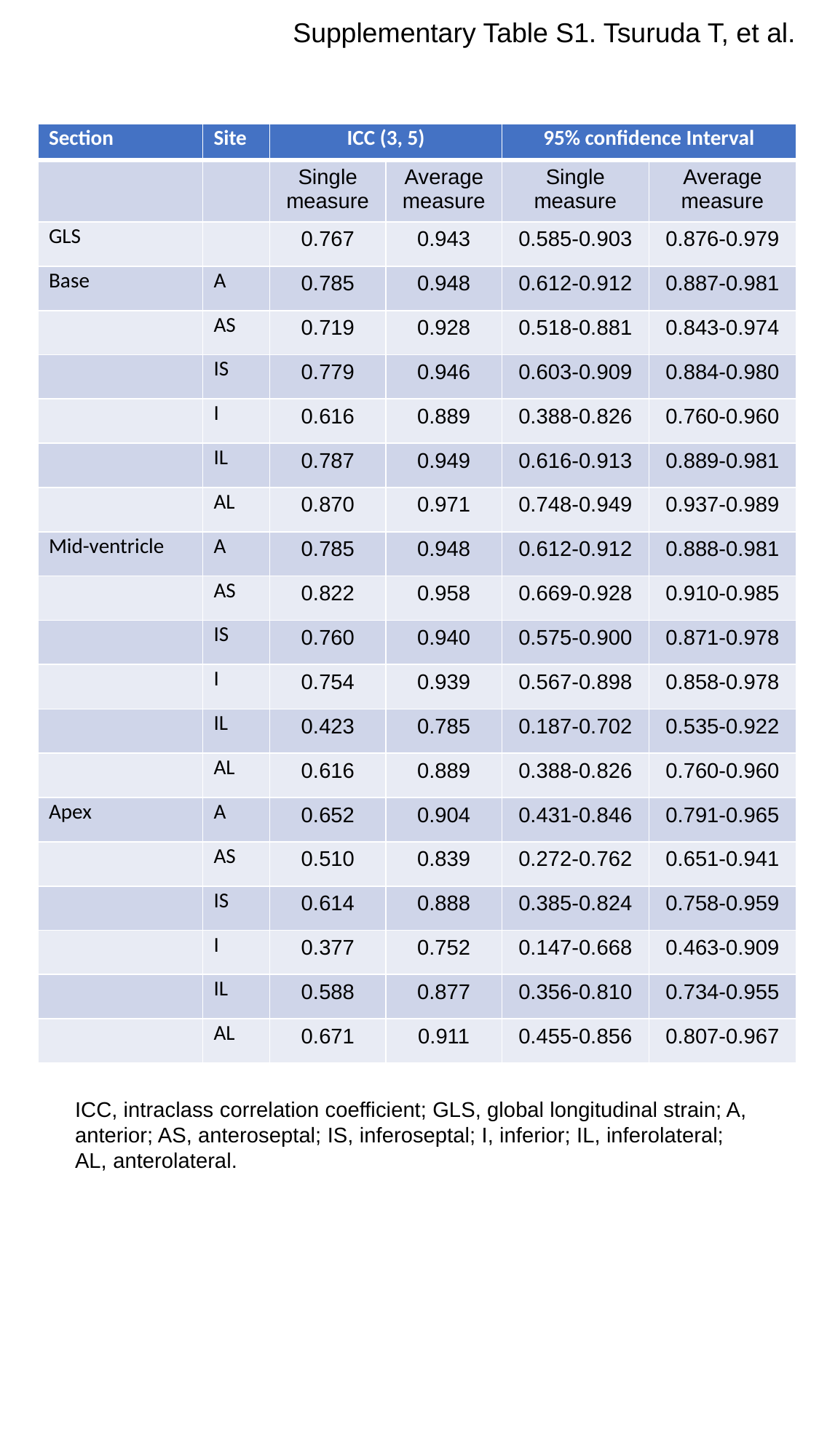

Supplementary Table S1. Tsuruda T, et al.
| Section | Site | ICC (3, 5) | | 95% confidence Interval | |
| --- | --- | --- | --- | --- | --- |
| | | Single measure | Average measure | Single measure | Average measure |
| GLS | | 0.767 | 0.943 | 0.585-0.903 | 0.876-0.979 |
| Base | A | 0.785 | 0.948 | 0.612-0.912 | 0.887-0.981 |
| | AS | 0.719 | 0.928 | 0.518-0.881 | 0.843-0.974 |
| | IS | 0.779 | 0.946 | 0.603-0.909 | 0.884-0.980 |
| | I | 0.616 | 0.889 | 0.388-0.826 | 0.760-0.960 |
| | IL | 0.787 | 0.949 | 0.616-0.913 | 0.889-0.981 |
| | AL | 0.870 | 0.971 | 0.748-0.949 | 0.937-0.989 |
| Mid-ventricle | A | 0.785 | 0.948 | 0.612-0.912 | 0.888-0.981 |
| | AS | 0.822 | 0.958 | 0.669-0.928 | 0.910-0.985 |
| | IS | 0.760 | 0.940 | 0.575-0.900 | 0.871-0.978 |
| | I | 0.754 | 0.939 | 0.567-0.898 | 0.858-0.978 |
| | IL | 0.423 | 0.785 | 0.187-0.702 | 0.535-0.922 |
| | AL | 0.616 | 0.889 | 0.388-0.826 | 0.760-0.960 |
| Apex | A | 0.652 | 0.904 | 0.431-0.846 | 0.791-0.965 |
| | AS | 0.510 | 0.839 | 0.272-0.762 | 0.651-0.941 |
| | IS | 0.614 | 0.888 | 0.385-0.824 | 0.758-0.959 |
| | I | 0.377 | 0.752 | 0.147-0.668 | 0.463-0.909 |
| | IL | 0.588 | 0.877 | 0.356-0.810 | 0.734-0.955 |
| | AL | 0.671 | 0.911 | 0.455-0.856 | 0.807-0.967 |
ICC, intraclass correlation coefficient; GLS, global longitudinal strain; A, anterior; AS, anteroseptal; IS, inferoseptal; I, inferior; IL, inferolateral; AL, anterolateral.
